# Necrostatin-1 Ameliorates Neutrophilic Inflammation in Asthma by Suppressing MLKL Phosphorylation to Inhibiting NETs Release

**DOI:** 10.3389/fimmu.2020.00666

**Published:** 2020-04-24

**Authors:** X. A. Han, H. Y. Jie, J. H. Wang, X. M. Zhang, Jun Wang, C. X. Yu, J. L. Zhang, J. He, J. Q. Chen, K. F. Lai, E. W. Sun

**Affiliations:** ^1^Department of Rheumatology and Immunology, The Third Affiliated Hospital, Southern Medical University, Guangzhou, China; ^2^Guangdong Provincial Key Laboratory of Bone and Joint Degeneration Diseases, The Third Affiliated Hospital, Southern Medical University, Guangzhou, China; ^3^Department of Respiratory and Critical Care Medicine, The Third Affiliated Hospital, Southern Medical University, Guangzhou, China; ^4^Department of Respiration, Nan Fang Hospital, Southern Medical University, Guangzhou, China; ^5^Department of Rheumatology and Immunology, Peking University Shenzhen Hospital, Shenzhen, China; ^6^Department of Rehabilitation Medicine, The Third Affiliated Hospital, Southern Medical University, Guangzhou, China; ^7^State Key Laboratory of Respiratory Disease, Guangzhou Institute of Respiratory Disease, The First Affiliated Hospital, Guangzhou Medical College, Guangzhou, China

**Keywords:** neutrophilic inflammation, neutrophil extracellular traps, Necrostatin-1, MLKL, asthma

## Abstract

Neutrophilic inflammation occurs during asthma exacerbation, and especially, in patients with steroid-refractory asthma, but the underlying mechanisms are poorly understood. Recently, a significant accumulation of neutrophil extracellular traps (NETs) in the airways of neutrophilic asthma has been documented, suggesting that NETs may play an important role in the pathogenesis. In this study, we firstly demonstrated that NETs could induce human airway epithelial cell damage *in vitro*. In a mouse asthmatic model of neutrophil-dominated airway inflammation, we found that NETs were markedly increased in bronchoalveolar lavage (BAL), and the formation of NETs exacerbated the airway inflammation. Additionally, a small-molecule drug necrostatin-1 (Nec-1) shown to inhibit NETs formation was found to alleviate the neutrophil-dominated airway inflammation. Nec-1 reduced total protein concentration, myeloperoxidase activity, and the levels of inflammatory cytokines in BAL. Finally, further experiments proved that the inhibition of Nec-1 on NETs formation might be related to its ability to inhibiting mixed lineage kinase domain-like (MLKL) phosphorylation and perforation. Together, these results document that NETs are closely associated with the pathogenesis of neutrophilic asthma and inhibition of the formation of NETs by Nec-1 may be a new therapeutic strategy to ameliorate neutrophil-dominated airway inflammation.

## Introduction

Asthmatic inflammation is dominated by the accumulation of eosinophils, neutrophils, or both, in the airways. Approximately half of the patients with asthma have eosinophilic inflammation, while the rest are characterized by increased number of neutrophils in sputum (1). Eosinophilic inflammation is commonly considered as type 2 inflammation and sensitive to inhaled corticosteroids, while Neutrophilic inflammation in asthma occurs in the absence of Th2 cytokines IL-4, IL-5, and IL-13, and insensitive to inhaled corticosteroids (2). In patients with acute or persistent asthma, the number of neutrophils is increased and correlates with a poor response to inhaled corticosteroids (3, 4). In adult asthma patients, airway neutrophil counts are associated with the severity of the disease (5, 6). Indeed, neutrophils have been demonstrated to play a critical role in airway inflammation in patients with acute and severe asthma (5, 7, 8). Therefore, neutrophil-predominant inflammation frequently occurs during asthma exacerbation, or in steroid-refractory patients, while the molecular mechanisms has not been determined.

Effective immune regulation depends on the balance between cell proliferation and death (9). Neutrophilic asthma is characterized by massive influx of neutrophils into the airway. Delayed apoptosis and prolonged survival of neutrophils are the potential mechanisms contributing to the persistent airway neutrophilia in severe asthma (10). Surprisingly, although glucocorticoids are usually recognized as anti-inflammatory, administration of glucocorticoids to neutrophilic asthmatic patients aggravates lung inflammation, since glucocorticoids augment the survival of neutrophils by delaying their apoptosis and ultimate clearance from lung tissues (11). Several therapies targeting neutrophils had been developed for the treatment of neutrophilic asthmatic patients, such as a CXCR2 antagonist (12), TNF-a antagonist (13–15), and a human anti-IL-17A monoclonal antibody (16). However, these therapies usually result in unsatisfactory responses or appear to cause mechanism-related side-effects, limiting their clinical application. Thus, the necessity of developing novel effective treatments for neutrophilic asthma is appealing.

Neutrophils constitute the first line defense during pulmonary infection, by efficiently binding, engulfing, and killing pathogens through phagocytosis, degranulation, and specifically release of neutrophil extracellular traps (NETs) (17). NETs are web-like structures comprising DNA and antimicrobial proteins, ejected from neutrophils undergoing a specific form of cell death called NETosis (18). Recently, extracellular DNA traps have been identified in both eosinophilic and neutrophilic asthmatic airways (19). However, subjects with neutrophilic asthma had higher neutrophil counts and NETs than eosinophil counts and eosinophil extracellular traps (EETs) (20). This difference is likely due to the fact that in neutrophilic asthma, especially in its severe form, neutrophil apoptosis is delayed, and clearance of the cell debris dysfunctioned, leading to an extensive accumulation of NETs (10, 11, 21). High extracellular DNA concentrations in sputum mark a subset of patients with more severe asthma (22, 23). NETs are generally believed critical for killing bacteria, but excessive NETs may be harmful (21, 24). So regulation of neutrophils releasing NETs may be a therapeutic target for neutrophilic inflammation in asthma.

Necrostatin-1 (Nec-1) has been reported as an inhibitor of the receptor-interacting protein 1 (RIP1) kinase and a potent and specific inhibitor of necroptosis (25, 26). Prior reports showed that Nec-1 could resolve inflammation by blocking RIP1 kinase activity in several experimental disease conditions, for example acute kidney injury, cardiac contractile dysfunction and brain injury after subarachnoid hemorrhage (27–29). Surprisingly, in our experiment, we unexpectedly found that Nec-1 inhibited NETs formation induced by phorbol 12-myristate 13-acetate (PMA). Therefore, in this study, we determined to investigate the exact mechanisms underlying this inhibition and its therapeutic effect on neutrophil-dominated asthma.

## Materials and Methods

This study was approved by the Ethics Committee of the Third Affiliated Hospital of Southern Medical University Trial Registration Identifier is at clinicaltrials.gov: NCT02385331.

### Subjects and Sputum Collection

Asthma was diagnosed by physicians based on the Global Initiative for Asthma (GINA) guidelines. We enrolled the asthmatics and chronic cough patients, who did not use systemic or inhaled corticosteroids for at least 4 weeks or more before sputum examination. Informed written consent forms were obtained from the participants, and the protocol was approved by the ethics committee of the Third Affiliated Hospital, Southern Medical University (2016–213). Sputum was induced using isotonic saline that contained a short-acting bronchodilator as previously study described (30). Sputum was prepared as follows: all portions with visibly greater solidity were carefully selected and placed in a pre-weighed Eppendorf tube. The total cell count was determined using a hema-cytometer. The sputum cells were collected by cytocentrifugation and 500 cells were examined after staining with Diff-Quick (American Scientific Products, Chicago, USA). Sputum samples that contained 10% squamous epithelial cells were excluded from the study. The remainder of the homogenized sputum sample was centrifuged at 1,000g for 5 min, and the supernatant was collected and stored at −70°C for subsequent protein analyses. Data here is from 11 adult asthma participants and 16 adult chronic participants who provided a sample of induced sputum.

### Neutrophil Isolation

Blood samples, 5 ml from each donor, were obtained from healthy donors. Neutrophils were isolated by dextran sedimentation and centrifugation as previously described (31). The cells were cultured in RPMI1640 medium containing 10% autologous serum.

### Induction, Isolation, and Quantification of NETs

The NETs were induced by PMA and isolated as previously described (32) with some modifications. Peripheral blood neutrophils (PBNs) were isolated from healthy donors, seeded onto six-well plates (9 × 10^6^ cells/well), and treated with 500 nM of PMA for 3 h. Supernatants were carefully suctioned and discarded, and neutrophils on the bottom of each dish washed by pipetting a total of 15 ml per dish of cold PBS in order to remove all adherent cells. Cells obtained from each dish were centrifuged for 10 min at 450 g at 4°C. Neutrophils and any remaining cells would pellet at the bottom, leaving a cell-free NET-rich supernatant. The supernatants containing cell-free DNA were collected and DNA concentrations quantified using Quant-iTTM PicoGreen dsDNA kit (Invitrogen, Paisley, UK) according to the manufacturer's instructions.

### Cell Culture and Cell Viability Assay

Human bronchial epithelial cell line 16HBE was donated by the Department of Pathophysiology, College of Life Sciences, Southern Medical University. The 16HBE cells were cultured in RPMI1640 medium containing 10% fetal calf serum. The cells were incubated in the absence or presence of various concentrations of NETs for 24 h and the cell viability tested by Cell Counting Kit (CCK-8) (Dojindo, Beijing, China) following the manufacturer's instructions. The concentration of extracellular lactate dehydrogenase (LDH) released by the neutrophils was measured using the LDH kit (Promega, USA). Cytotoxicity was calculated by the level of LDH released from the lysed neutrophils. The fraction of detached cells was calculated as (cell number in unwashed plates–cell number in the washed plates)/cell number in unwashed plates) × 100%. Level of the inflammatory cytokine IL-1β in the supernatant was assessed using an ELISA kit (R&D Systems, USA) according to the manufacturer's protocol.

### Immunofluorescence Analysis of NETs Formation *in vitro*

Human blood neutrophils were pretreated with or without Nec-1 for 30 min *in vitro*, placed on cytospin and stimulated with 25 nM PMA or PBS for 4 h. Subsequently, the cells were fixed with 1 ml PBS containing 1% paraformaldehyde. After blocking with 5% fetal bovine serum, the cytospin were stained with rabbit anti-MPO (Abcam, catalog ab208670, Cambridge, UK) followed by a secondary Cy3-conjugated goat anti-rabbit IgG antibody (Servicebio, catalog GB21303, Boston, MA, USA). The DNA was counterstained by DAPI (Thermo Fisher Scientific). The specimens were mounted and analyzed using a fluorescence microscope.

Hoechst 33342 staining and microscopy were used to confirm the presence of morphological characteristic of neutrophils. Neutrophils isolated from peripheral blood of healthy donors were seeded in six-well plates at 5 × 10^6^ cells/well and cultured for 3 h in 1,640 medium containing with 50 μM Nec-1, 25 nM PMA, or 50 μM Nec-1 together with 25 nM PMA. Subsequently, the cells were stained with Sytox Green and Hoechst 33342 for 15 min and analyzed by fluorescence microscopy.

Neutrophils isolated from peripheral blood of health donors were seeded in six-well plates at 5 × 10^6^ cells/well and the cells treated with 25 nM PMA or 50 μM Nec-1 together with 25 nM PMA for 30 min. FITC-marked non-membrane nucleic acid dye Sytox Green was used as DNA marker. Dynamic changes of neutrophil NETs after PMA stimulation were recorded in two channels, light field channel (measuring changes in cell morphology and structure under light microscope) and FITC green channel (measuring non-transmembrane nucleic acid dye Sytox Green. Imaging was performed with an automatic living cell imaging analysis system (Biotek Lionheart) in a temperature-controlled chamber at 36°C. Images (2,048 × 2,048 pixels) were acquired at 0.5% maximal laser intensity with the first image at PMA stimulation for 30 min and then at every 5 min for each well for a total of 4 h.

### Western Blotting

Western blot analysis was performed using isolated human neutrophil. Neutrophils (5 × 10^6^) were lysed with ice-cold RIPA (Radio Immunoprecipitation Assay) buffer containing a protease inhibitor cocktail for 30 min before centrifugation (14,000 rpm, 4°C, 5 min). All manipulations were performed on ice. Protein content was determined by using bicinchoninic acid (BCA) assay. Protein samples (30 μg per lane) were resolved by SDS-PAGE and then transferred to polyvinylidene difluoride (PVDF) membranes. Blots were blocked with 5% skimmed milk powder in TBS plus Tween before probing with antibodies to MLKL(ab194699, Abcam), pMLKL(ab187091,abcam) and β-actin(Sigma-Aldrich). The membranes were incubated overnight at 4°C with an anti- MLKL polyclonal antibody (1:1000 dilution), anti-PMLKL polyclonal antibody (1:1000 dilution), and anti-β-actin monoclonal antibody (1:5000 dilution; Sigma-Aldrich). Then they were incubated for 1 h at room temperature with an HRP-conjugated secondary antibody (1:5000 dilution; Thermo, Fremont, USA). The target protein was detected using an enhanced chemiluminescence solution (GE Healthcare, Little Chalfont, UK) on X-ray film.

### Experimental Animals

Wild-type male C57BL/6 mice, aged 6–7 weeks, were purchased from the Animal Center of the Southern Medical University and housed in a conventional animal facility. All protocols for the mice were approved by the Ethics Committee for Animal Studies at the Third Affiliated Hospital of Southern Medical University.

### Neutrophilic Inflammation Model of Murine Asthma

The neutrophil-dominated asthma model was established according to previously published protocols (30, 33). Mice were immunized by intraperitoneal injection of 20 μg grade V chicken egg ovalbumin (OVA; Sigma-Aldrich) at days 0 and 7. OVA was dissolved in endotoxin-free 25 μL of PBS emulsified in 75 μL of complete Freund's adjuvant (CFA; Sigma-Aldrich). On days 14 and 15, the immunized mice were challenged for 40 min with an ultrasonic nebulized (DeVilbis, Somerset, PA, USA) aerosol containing 0.1% OVA in saline (OVA/CFA group). Alternatively, the mice were challenged with the same aerosol in combination with a nasal drip of 30 μL of 25 nM PMA (OVA/CFA+PMA group). Mice assigned to the Nec-1 group were intraperitoneally injected Nec-1 (Sigma-Aldrich), 6 mg/kg body weight, 1 h after each OVA aerosol challenge. Control mice were intraperitoneally injected the same volume of saline. Twenty-four hours after the last OVA challenge, all mice were sacrificed and an autopsy performed. Following collection of BAL, the left lung was fixed in 4% paraformaldehyde, and the right lung was kept in tissue culture medium on ice before being homogenized for further processing.

### BAL Collection and Differential Cell Counts

BAL cells were collected by slowly injecting 0.5 mL of ice-cold PBS into the trachea using a 22-inch intravenous catheter and collecting the outflow fluids. The total cell number in BAL was counted with a Neubauer chamber. Additionally, cytospin specimen were prepared, stained with Wright-Giemsa, and a total of 400 cells counted and classified under a microscope to determine the fraction of eosinophils, neutrophils, lymphocytes, and macrophages. Results are expressed as the number of cells per milliliter of BAL.

### Evaluating the Formation of NETs

To quantify the amount of PMA-induced formation of NETs *in vitro*, neutrophils isolated from human blood (3 × 105 cells/well in 200 μL of medium) were seeded into black, flat-bottomed, 96-well plates and incubated for 4 h in a humidified incubator at 37°C with an atmosphere containing 5% of CO_2_. The medium was supplemented with phosphate-buffered saline (PBS), 50 or 100 μM Nec-1, and with or without 25 nM PMA. Extracellular DNA was stained with the membrane-impermeable DNA-binding dye Sytox Green (Thermo Fisher Scientific, USA). The plates were analyzed using Spectra Max M3 fluorescent plate reader (Molecular Devices) with excitation at 485 nm and emission at 520 nm. The same approach was employed to measure the level of NETs in the BAL. For this purpose, 150 μL of BAL per well was used.

### Flow Cytometric Analysis of BAL Cells

BAL cell suspensions were obtained directly from the mice or from *in vitro* cultures. The expression of cell surface markers was determined by flow cytometry using fluorescent dye-conjugated mouse antibodies. Mouse neutrophils were identified by Ly6G expression. To analyze apoptosis, BAL cells were incubated with FITC-labeled annexin V and propidium iodide (Multi-Sciences, Hangzhou, China). Data were acquired with the FACSVerse™ (BD Biosciences) and analyzed with the FlowJo software (version 7.6).

### Immunofluorescent Staining of the BAL Cells

The BAL was collected from mouse lungs 24 h after the final challenge. The BAL cells were incubated on poly-L-lysine-coated coverslips for 4 h. Subsequently, the liquid was discarded from the coverslips and the cells fixed for 15 min in 1 ml PBS/ 1% paraformaldehyde. The fixed cells were then washed with ice-cold PBS and blocked for 90 min with 5% fetal bovine serum. Next, the cells were incubated with the primary antibody, anti-myeloperoxidase (MPO) or anti-cleaved caspase3 (Cleave-caspase3). Following washing, samples were incubated for 45 min with an Alexa 568-labeled (goat anti-rabbit) or an Alexa Fluor 488-labeled secondary antibodies (Donkey anti-goat). Coverslips were mounted onto glass slides using ProLong Gold mounting medium (Thermo Fisher Scientific, USA), sealed, and analyzed by fluorescence microscopy.

### Evaluation of NETs in BAL Fluid

BAL was collected from mouse lungs 24 h after the final challenge and centrifuged at 1,000 rpm for 10 min. To detect NETs, 150 μL of supernatant was mixed with 1.5 μL Sytox Green dye (Thermo Fisher Scientific, USA) that was diluted 1:50 in PBS.

### Histopathologic Evaluation of Lung Tissue

To maintain tissue integrity, mice to be used for histologic evaluation were not subjected to bronchoalveolar lavage. Lungs were fixed in 10% formalin and embedded in paraffin. Sections were cut, stained with hematoxylin and eosin, and observed under a microscope. All slides were examined independently by two investigators blinded to the experimental treatment.

### Immunohistochemistry of Lung Tissue

Lungs were removed, fixed in 4% paraformaldehyde for 4 h, and immersed in 30% sucrose for 3–4 days at 4°C. Sections, 15 μm thick, were cut from frozen lung tissues on a cryostat. Rabbit anti-cleaved caspase-3 (Asp175, diluted 1:500 in PBS; Cell Signaling Technology) and mouse anti-MPO (ab90810; diluted 1:100 in PBS; Abcam) were used as primary antibodies. The staining was visualized with a confocal laser scanning microscope (Leica DM IRE2, Germany). MPO and cleaved caspase-3 were used to identify, neutrophils and apoptotic cells, respectively. Double-positive cells were considered to represent apoptotic neutrophils.

### Measurement of Airway Hyperresponsiveness

Airway responsiveness was determined with a non-invasive method to measure mouse lung resistance after the mice were challenged with aerosolized methacholine (Sigma-Aldrich). Briefly, 24 h after the final OVA challenge, mice were exposed to an aerosol containing increasing concentrations of methacholine (0, 1.5625, 3.125, 6.25, 12.5, and 25 mg/mL in saline) using Buxco FinePointe plethysmograph (Buxco Electronics, Troy, NY). Data on lung resistance were collected continuously, and mean values represented changes in airway function.

### Measurement of Pro-inflammatory Cytokines in BAL

The levels of inflammatory cytokines TNF-α, IL-1β, and IFN-γ were measured with ELISA kits (R&D Systems, USA) according to the manufacturer's protocol.

### Measurement of MPO

Lung tissues were frozen in liquid nitrogen and homogenized in PBS. The homogenate was used to determine MPO according to the manufacturer's Instruction (Nanjing Jian Cheng Bioengineering Institute, China).

### Total Protein Concentration in BAL

Total protein concentration in the supernatant of BAL was measured by the Bradford method with bovine serum albumin (Beyotime Institute of Biotechnology, China) used as the standard.

### Preparation of Nec-1

Nec-1 was purchased from Sigma-Aldrich; 5 mg of the compound were dissolved in 0.5 ml DMSO, and 100 μL aliquots were stored at −20°C.

### Statistical Analysis

Results are presented as means ± SEM. Data were analyzed with one-tailed Student *t*-test (1-tailed) or one-way ANOVA, followed by the Tukey *post-hoc* test or the non-parametric Wilcoxon rank-sum test. All statistical analyses were performed using the Prism7 software (GraphPad Software, La Jolla, CA, USA). *P* < 0.05 was considered statistical significance.

## Results

### Sputum eDNA Levels Are Significantly Correlated With Sputum Neutrophils in Asthmatics and Chronic Cough Patients, and NETs Induced Damage of Human Airway Epithelial Cells

**Figure 1:**
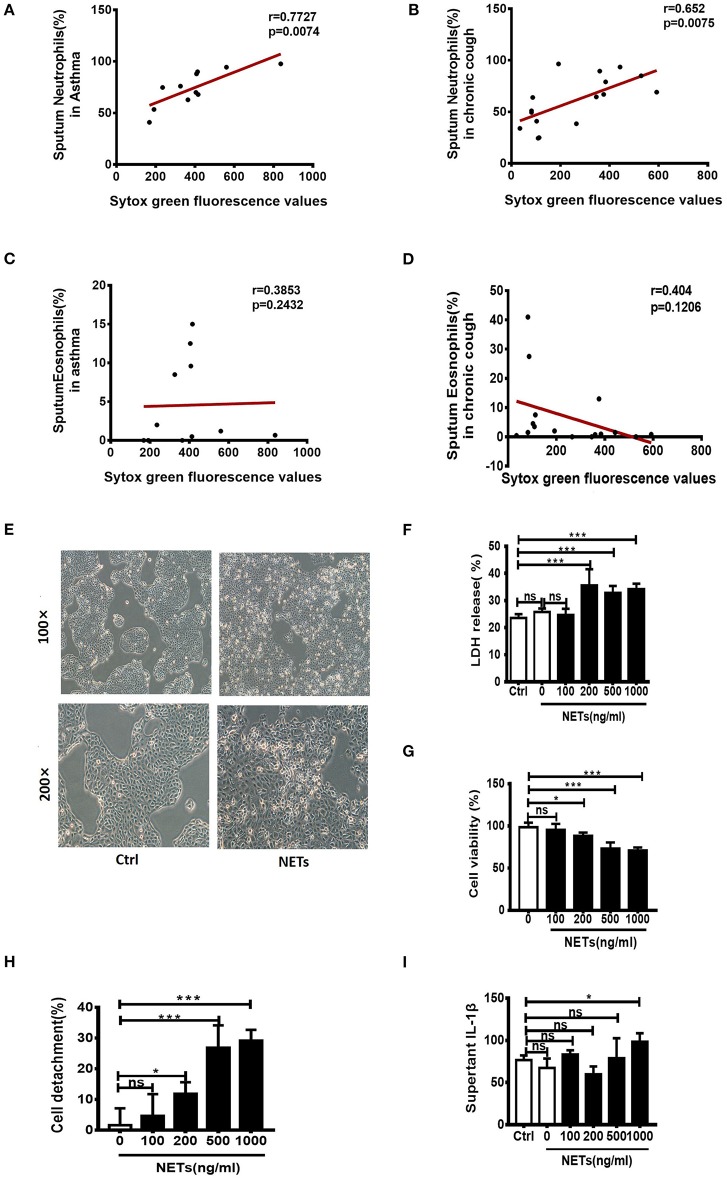
Sputum extracellular DNA (eDNA) levels are significantly correlated with sputum neutrophils in asthmatics and chronic cough patients, and neutrophil extracellular DNA traps (NETs) induced damage of human airway epithelial cells. **(A,B)** Extracellular DNA in sputum stained with Sytox Green. Sputum eDNA levels were significantly correlated with sputum neutrophils percentage in asthmatics and chronic cough. **(C,D)** Sputum eDNA levels were not significantly correlated with sputum eosinophil percentage in asthmatics and chronic cough. **(E)** Morphological changes of 16HBE cells after treatment with 500 ng/ml of NETs for 24 h. 16HBEs were treated with different concentrations of NETs for 24 h, and LDH release **(F)**, cell viability **(G)**, detached cells (%) **(H)** and supernatant interleukin (IL)-1β **(I)** were detected. The data are shown as mean ± SD. (*p < 0.05, ***p <0.001, ns, not significant). The results are representative of at least three experiments.

It has been reported that sputum extracellular DNA (eDNA) in asthma is associated with airway neutrophilic inflammation, and increases in soluble NET components. In the study firstly we detected the levels of sputum eDNA in asthma and chronic cough patients. Sputum samples of 11 patients with asthma and 16 patients with chronic cough were collected. We found that sputum eDNA levels were significantly correlated with sputum neutrophil percentage in asthmatics and chronic cough patients (Figures 1A,B), but not significantly correlated with sputum eosinophil percentage in asthmatics and chronic cough patients (Figures 1C,D). Although excessive NETs are present in the airways of neutrophil-dominate inflammation, whether they have direct effect on bronchial epithelial cells is unclear. Therefore, different concentrations of NETs were co-cultured with human bronchial epithelial cell line 16HBEs for 24 h. The morphology of the cells changed significantly, from a triangle or polygon to a round shape, and the cells detached from the bottom of the culture plate (Figure 1E). Cell death rate and extracellular level of LDH were significantly increased when the concentrations of NETs were more than 200 ng/mL (Figure 1F). The impact of NETs on cell viability and detachment of cells were dose-dependent (Figures 1G,H). Moreover, the inflammatory cytokine IL-1β in the culture supernatant was significantly increased by the treatment of 1,000 ng/mL of NETs (Figure 1I). Thus, NETs induce damage of airway epithelial cells and trigger an inflammatory response.

### NETs Aggravated Neutrophil-Dominated Airway Inflammation

**Figure 2:**
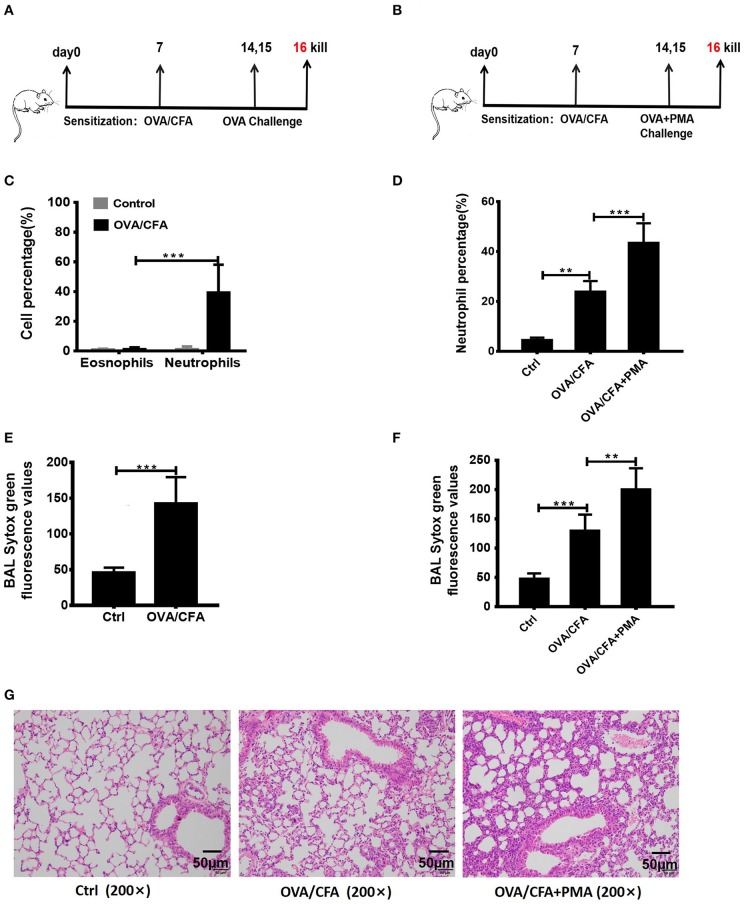
NETs aggravated neutrophil-dominated airway inflammation. **(A)** Schematic diagram of OVA/CFA sensitized neutrophil-dominated airway inflammation model. **(B)** Schematic diagram of OVA/CFA plus PMA sensitized neutrophil-dominated airway inflammation model. **(C)** Pulmonary inflammatory cell populations of OVA/CFA sensitized mice with neutrophil-dominated airway inflammation. **(D)** The percentages of neutrophils in total BAL cells of OVA/CFA and OVA/CFA plus PMA groups. **(E,F)** Extracellular DNA in BAL stained with Sytox Green. **(G)** Representative images of H&E-stained lung tissues of OVA/CFA and OVA/CFA plus PMA groups. Values are expressed as means ± SEM of three independent experiments. (**p < 0.01, ***p < 0.001).

To determine whether NETosis and associated extracellular DNA contribute to the pathogenesis of neutrophilic asthma, the OVA/CFA induced neutrophil-dominated asthma-like airway inflammation model was employed (33), according to the schemes illustrated in Figure 2A. As expected, a substantial infiltration of neutrophils in the airways is found in the OVA/CFA induced mouse models (Figure 2C). Staining with Sytox Green revealed that the NETs in the BAL were significantly increased (Figure 2E). PMA is a strong inducer of NETs formation, therefore PMA (25 nM) was administered intranasally to induce NETs in the airways of OVA/CFA-induced mice (Figure 2B). Exposure of mice to PMA resulted in a significant increase of neutrophils and NETs in BAL compared with model mice (Figures 2D,F). Additionally, histologic examination of the lungs documented that airway inflammation was more severe in the PMA-stimulated group than OVA/CFA alone group (Figure 2G). The data suggest that NETs are involved in the pathogenesis of neutrophilic asthma and accumulation of NETs may aggravate neutrophil-dominated airway inflammation.

### Nec-1 Significantly Inhibited PMA-Induced NETs Formation *in vitro*

**Figure 3:**
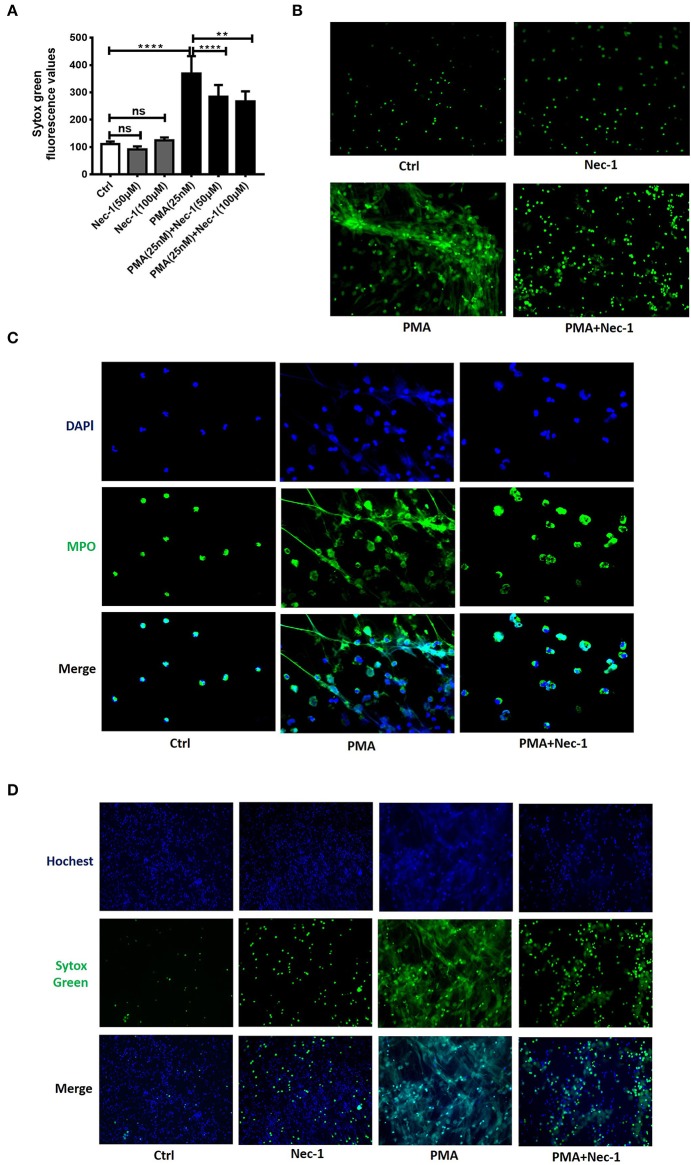
Nec-1 inhibited PMA-induced NETs formation *in vitro*. **(A)** Levels of extracellular DNA released by peripheral blood neutrophils, which were cultured with PMA (25 nM) or different concentrations of Nec-1 (50 or 100 μM), or both. **(B)** Representative immunofluorescence images of Sytox Green-stained neutrophils cultured for 3 h in the absence (ctrl) or presence of PMA (25 nM), Nec-1 (50 μM), or both (200**×**). **(C)** Representative immunofluorescence images of neutrophils pretreated with Nec-1 (50 μM) and stimulated with or without PMA (25 nM). Upper panels illustrate DAPI (blue), central panels illustrate MPO (green), lower panels show merged images (400**×**). **(D)** Representative immunofluorescence images of neutrophils pretreated without (ctrl) or with Nec-1(50 μM) and stimulated with PMA (25 nM) or both. Upper panels illustrate Hoechst-33342 (blue), central panels illustrate Sytox Green, and lower panels show merged images (200×). The values were shown as mean ± SEM. (**p < 0.01, ****p < 0.0001, ns, not significant). The results are representative of three independent experiments.

Nec-1 has been recognized as a potent and specific inhibitor of necroptosis. To investigate whether Nec-1 inhibited PMA-stimulated NETs formation, peripheral blood neutrophils (PBNs) isolated from healthy donors and cultured for 3 h in the absence (control) or presence of different concentration of Nec-1 (50 or 100 μM), with or without PMA (25 nM). The results demonstrated that Nec-1 significantly inhibited PMA-induced NETs formation (Figure 3A) with representative images shown in Figure 3B. Immunofluorescent staining of MPO (a marker of neutrophils) and DAPI (staining of nuclei) further documented the inhibitory effect of Nec-1 (Figure 3C, Figure S4). The effect of Nec-1 on neutrophil releasing NETs was also examined by staining with Sytox Green and Hochest-33342. And in comparison with the group stimulated by PMA alone, less fluorescence of Sytox Green was present in cells treated with PMA (25 nM) plus Nec-1(50 μM) (Figure 3D). Through these experiments confirmed that Nec-1 inhibited PMA-stimulated NETs formation, but the exact mechanism was unclear. As Nec-1 could inhibit PMA-induced NETs formation and the amount of NETs corresponded to the damage of airway bronchial epithelial cells, we believed that Nec-1 could be used to treat neutrophil-dominated airway inflammation.

### Nec-1 Attenuated OVA/CFA-Induced Neutrophil-Dominated Airway Inflammation by Inhibiting NETs Formation

**Figure 4:**
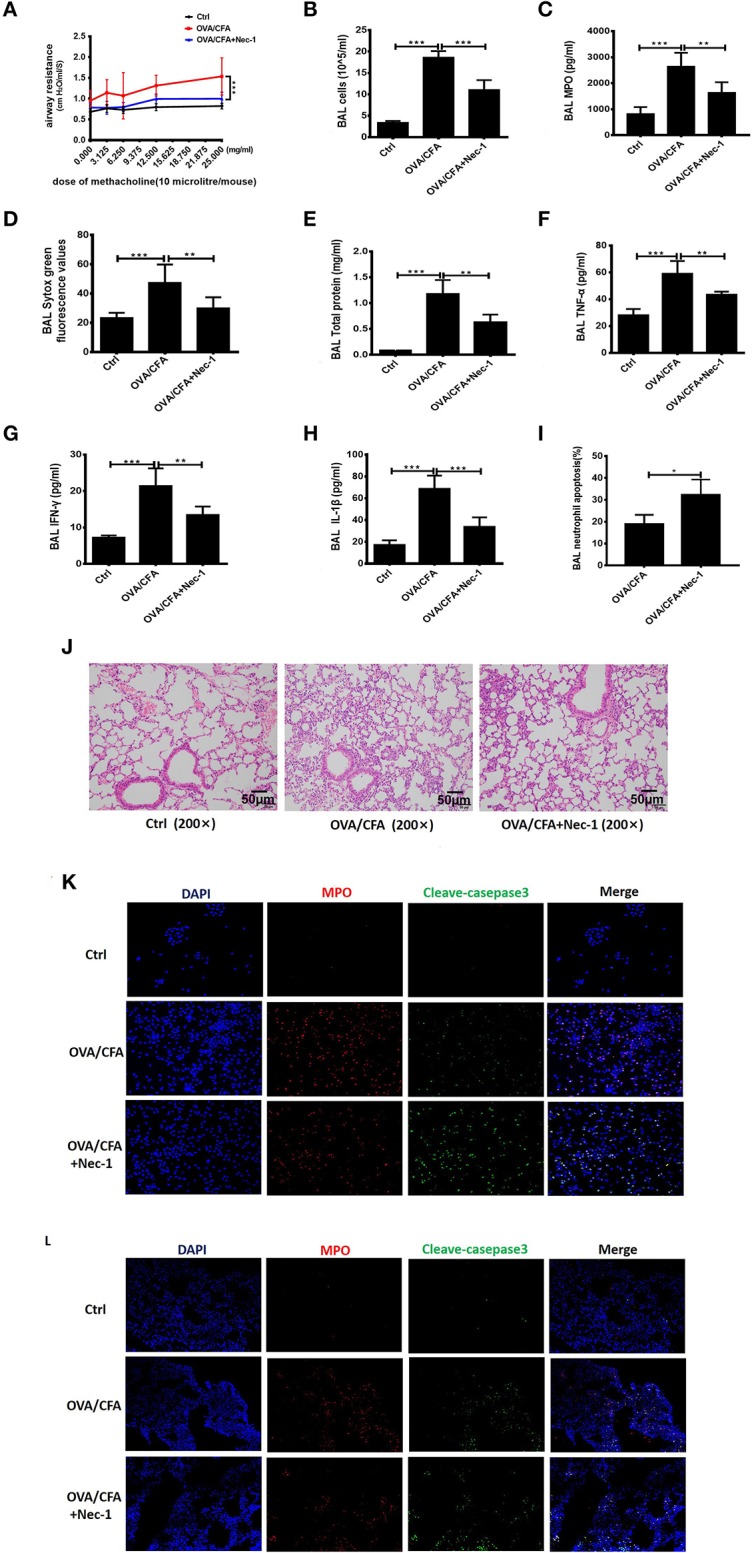
Nec-1 attenuated OVA/CFA sensitized neutrophil-dominated airway inflammation by inhibiting NETs formation. OVA/CFA sensitized mice were treated with Nec-1 (6 mg/kg body weight), and BAL harvested. **(A)** OVA/CFA sensitized mice were challenged with different doses of methacholine and treated with Nec-1 (6 mg/kg body weight), and BAL and lung tissues were harvested. Airway hyper-responsiveness used to assess the responses of mice to different doses of methacholine. **(B)** Total number of cells in BAL. **(C)** MPO activity in lung tissue homogenate. **(D)** Levels of extracellular DNA in BAL stained with Sytox Green. **(E)** Total protein concentration in BAL. **(F–H)** Levels of TNF-α, IFN-γ, and IL-1β in BAL. **(I)** Fraction of apoptotic neutrophils in the BAL determined by flow cytometry. Quantitative analysis of flow cytometry data (*n* ≥ 3; ***P < 0.001 vs. control). **(J)** Representative images of H&E-stained lung tissue of mice from different experimental groups. **(J)** Representative immunofluorescence images of BAL cells. From left to right, the panels illustrate staining with DAPI, MPO antibody, and cleaved caspase-3 antibody. The right panels show the merge of images (200×). **(K,L)** Representative immunofluorescence images of BAL cells and lung tissues of control, OVA/CFA, and Nec-1 groups (200×). From left to right, the panels illustrate staining with DAPI, MPO antibody, and cleaved caspase-3 antibody. The right panels show the merge of image. Values are expressed as means ± SEM. (*p < 0.05, **p < 0.01, ***p < 0.001, ns, not significant).

To test whether Nec-1 can be used in the treatment of neutrophilic asthma, OVA/CFA neutrophil-dominated airway inflammation mouse model was established (shown in Figure 2A). Airway hyper-responsiveness (AHR), assessed by a non-invasive ventilator, was alleviated in Nec-1 treatment group (Figure 4A). Furthermore, we tested level of NETs in the BAL fluid (Figure 4B). Sytox Green staining revealed that the level of NETs in the BAL from OVA/CFA primed mice was reduced by Nec-1 treatment (Figure 4D). Other inflammatory indicators such as total protein concentration (an indicator of lung leakage), pulmonary MPO activity (a marker of neutrophils), and pro-inflammatory mediators IL-1β, TNF-α, and IFN-γ in BAL were significantly decreased in the Nec-1 treatment group (Figures 4C,E–H). Histological examination also showed that the lung inflammation was markedly reduced by Nec-1 (Figure 4J). Thus, we speculated that Nec-1 could resolve neutrophil-dominated airway inflammation by inhibiting NETs release, but the exact mechanism underlying the inhibition of NETs formation was unclear.

Furthermore, we also found neutrophil apoptosis increased *in vivo* study. Neutrophil apoptosis in the BAL was analyzed by flow cytometry. Neutrophils were labeled by Ly6G antibody and apoptosis was distinguished from necrosis by FITC-labeled Annexin V and PI. In BAL of OVA/CFA primed mice, annexin V-positive neutrophils increased significantly (*p* < 0.001) by Nec-1 treatment (Figure 4I). Additionally, immunofluorescence analysis of BAL cells demonstrated that Nec-1 treatment group was associated with a decreased number of MPO-positive cells and an increased number of cleaved-caspase 3-positive cells (Figure 4K). Also, immunofluorescence histochemistry assay of lung tissue also showed that Nec-1 treatment group had less MPO positive cells and more cleaved-casepase3 positive neutrophils compared with OVA/CFA group (Figure 4L). This *in vivo* study further confirmed that Nec-1 reduced NETs formation and induced neutrophil apoptosis in both BAL cells and lung tissue. Thus, *in vitro* and *in vivo* studies support the notion that Nec-1 inhibits NETosis while promoting apoptosis.

### Nec-1 Inhibited PMA-Induced NETs Formation by Suppressing Phosphorylation and Perforation of MLKL *in vitro*

**Figure 5:**
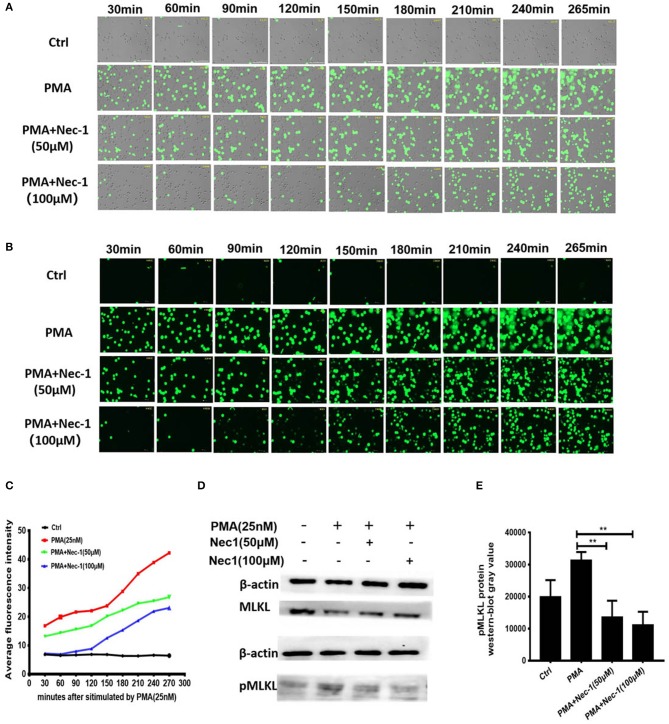
Nec-1 inhibited PMA-induced NETs formation by suppressing phosphorylation and perforation of MLKL *in vitro*. **(A,B)** Sytox Green stained neutrophils, stimulated with 25 nM PMA alone, or with different concentrations of Nec-1 (50 or 100 μM) and imaged for 4 h using an automatic live cell imaging analysis system (Videos S1–S4 respectively indicated group Ctrl,PMA (25 nM), PMA+Nec-1 (50 μM) and PMA+ (100 μM) were shown in additional files). **(A)** showed light field channel (measuring changes in cell morphology and structure under ordinary optics microscope). **(B)** showed FITC green channel (measuring non-transmembrane nucleic acid dye Sytox Green). The green fibrin-like structure indicated NETs. **(C)** The mean fluorescence intensity of Sytox Green at different time points analyzed by Image J semi-quantitative statistics, and the trends of NETs after PMA stimulation analyzed, as shown in **Video** is representative of three independent experiments. Values are expressed as means ± SEM of three independent experiments (*p < 0.05, **p < 0.01, ***p < 0.001, ns, not significant). **(D)** Human neutrophils (5 × 10^6^ cells/ml) were treated with buffer control, PMA (25 nM) with or without different concentrations of Nec-1 (50 or 100 μM) for 2 h. After cell lysis, proteins were subjected to MLKL and pMLKL. Western Blots are representative of at least three separate experiments. **(E)** Westerblot results of three times with grayscale analysis and statistical analysis (**p < 0.01).

In order to explore the exact mechanism underlying the inhibition of NETs formation of Nec-1, we used an automatic living cell imaging analysis system to capture the morphological changes of the cells that were stained with cell impermeable stain Sytox Green (Videos S1–S4 indicated different groups were shown in Supplementary Files). We found that in PMA stimulation group the nuclei of neutrophils first condensed and cells rapidly depolymerized and swelled, and then the nuclei disintegrated and cell membrane collapsed. These results suggest that PMA induces degradation of DNA and pore formation in the cytoplasmic membrane, which results in membrane rupture and release of a large number of NETs. Interestingly, Nec-1 does not inhibit nuclear shrinkage, cell swelling, or chromosome depolymerization, but it dependently decreased the number of cells with membrane rupture and release of NETs (stained with Sytox Green) (Figures 5A–C). It is suggested that Nec-1 inhibits the production of NETs by protecting the integrity of cell membrane. Prior study indicated that Nec-1 selectively targets the kinase activity of RIPK1 and involved regulating RIPK3/ MLKL-dependent necroptotic death (26), so we further study the downstream molecules of RIPK1. Western-blotting experiments showed that PMA could cause MLKL phosphorylation, but Nec-1 inhibited mixed lineage kinase domain-like (MLKL) phosphorylation (Figures 5D,E, and Figure S1). As we know after phosphorylation, MLKL form a polymer to punch holes in the cell membrane and disrupt it (34, 35). Therefore, we believe that Nec-1 inhibits neutrophil release of NETs by suppressing phosphorylation and perforation of MLKL.

## Discussion

Neutrophilic asthma is characterized by a massive influx of neutrophils into the airways, which has been associated with persistent airway inflammation (36, 37). Although infiltration of excess neutrophils is thought to aggravate pulmonary inflammation, the detailed mechanism remains unclear. NETosis is a specific form of cell death of neutrophils that is distinct from apoptosis and other types of necrosis by releasing large web-like NETs (38). NETs are composed of DNA strands associated with histones and decorated with lots of different proteins. Although NETosis and the released NETs could provide important biological advantage for the host to fight against certain microbial infections (39). NETosis does not only occur during infection, but is also initiated by proinflammatory cytokines such as IL-8, TNF-α, platelet-activating factor (PAF), and GM-CSF, as well as other stimulants such as LPS, PMA and complement factor 5a (C5a) (40). However, NETs act as double-edged swords, high levels of NETs lead to more significant release of proinflammatory cytokines, further accelerating inflammation (20, 21, 41). The presence of a large amount of NETs in the airways of neutrophilic asthma has been suggested in the pathogenesis and exacerbation of neutrophilic asthma (22–24). In our study we first demonstrated that sputum eDNA levels are significantly correlated with sputum neutrophils in asthmatics and chronic cough, but not significantly correlated with sputum eosinophils (Figures 1A–D). To determine the role of NETs in neutrophil-dominated asthma, in this study, we further assessed whether isolated NETs could damage airway epithelial cells *in vitro*. We found that NETs directly caused death of airway epithelial cells and resulted in the cells to release a large amount of inflammatory factors (Figures 1E–I). We also used dsDNA synthesized *in vitro*, and found it damage vein endothelial cells (Figure S2). Therefore, the NETosis and NETs release should be tightly regulated to avoid damage to the host.

To further study the role of NETs in asthma, a classic OVA/CFA induced allergic pulmonary inflammation model was selected (23, 26). The OVA/CFA model is mainly manifested by neutrophil infiltration, poor response to corticosteroid (42), and the participation of Th1 and Th17 cells. The mice were OVA sensitized in the presence of complete Freund's adjuvants (CFA), a potent Th-1/Th-17-skewing adjuvant commonly used in mouse models of hypersensitivity pneumonitis, and followed by OVA aerosol challenge. During current experiments, a large number of NETs were detected in the airway of OVA/CFA model mice (Figure 2E), suggesting that NETs may be closely associated with the onset of neutrophil-dominated allergic pulmonary inflammation. To test the possibility that increased NETs release leaded to the aggravation of neutrophilic asthma, PMA, a potent inducer of NETosis, was administered intranasally. High levels of NETs in the BAL was found and significantly correlated with the proportion of neutrophils in BAL and the severity of airway inflammation (Figure 2). This result demonstrates that NETs constitute one of the important factors causing progression and exacerbation of neutrophilic asthma. Both *in vitro* and *in vivo* studies have shown that NETs can cause epithelial injury in the airway and promote the occurrence and development of neutrophil-dominated asthma. Thus, accumulated data indicate that NETs can become a potential target for the treatment of neutrophil-dominated asthma. To harness the power of NETs while producing minimal damage, it is critical to maintain the right abundance of NETs formation, but reduce the accumulation of excessive NETs to avoid tissue damage.

Nec-1 was identified as a classic inhibitor of necroptosis (26, 43). It selectively targets the activity of receptor-interacting protein 1 kinase (RIPK1) that regulates the receptor-interacting protein 3 kinase/mixed-lineage kinase domain-like protein (RIPK3/MLKL)-dependent necroptotic death of mouse and human neutrophils. Almost all studies on Nec-1 were mainly focused on its role of the inhibition of necroptosis (43, 44). In our study, we found that Nec-1 inhibited PMA-induced NETs formation (shown in Figure 3, Figure S4). Our *in vivo* study showed that Nec-1 could alleviate airway hyperresponsiveness in OVA/CFA mouse model (Figure 4A), and also reduced the level of NETs, total protein concentration and MPO activity in BAL as well (Figures 4C–E). The levels of inflammatory factors including IL-1β, IFN-γ, and TNF-α in BAL were also reduced (Figures 4F–I). Histologic examination documented the reduction of inflammation in OVA/CFA induced neutrophil-dominated asthma in Nec-1 treated mice (shown in Figure 4J), suggesting that administration of Nec-1 can alleviate airway inflammation in neutrophil-dominated asthma by inhibiting the formation of NETs.

In order to explore the exact mechanism of Nec-1 inhibited NETs, the automatic live cell imaging analysis system was used to further dynamically capture the morphological changes of the cells. Interestingly, we found that although Nec-1 could not inhibit nuclear shrinkage, cell swelling and chromosome depolymerization caused by PMA, it could inhibit cell membrane rupture of neutrophils and protect the integrity of cell membrane (Figures 5A–C). And we further studied the downstream molecules of RIPK1. Western-blotting experiments showed that Nec-1 inhibited mixed lineage kinase domain-like (MLKL) phosphorylation (Figures 5D,E, Figure S1). Prior study indicated that the necroptotic cell death effector MLKL phosphorylation forming a polymer which translocated from the cytoplasm to the plasma membrane to perforation (45–48). Prior study indicated that MLKL phosphorylation could stimulate downstream NADPH oxidase-independent ROS production, breakdown of the nuclear membrane, and induce NETs formation (49). So we speculate that Nec-1 suppresses the release of NETs by inhibiting phosphorylation and perforation of MLKL.

**Figure 6:**
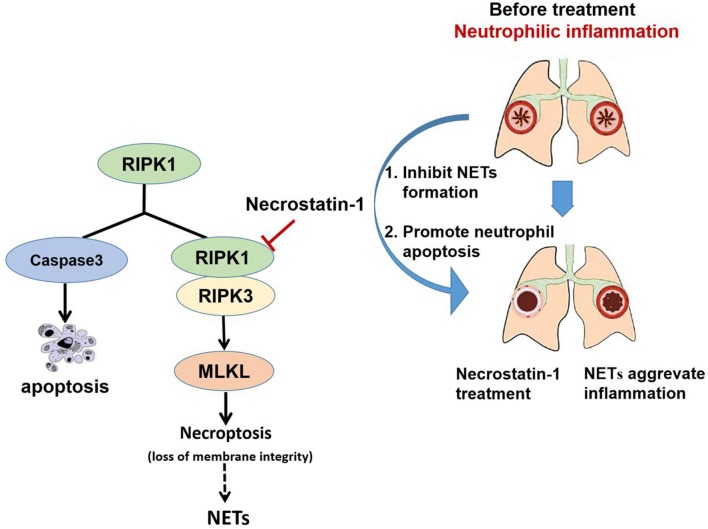
Schematic illustration of Necrostatin-1(Nec-1) ameliorates neutrophilic inflammation by suppressing MLKL phosphorylation to inhibiting NETs release. As a necroptosis inhibitor, Nec-1 selectively targets the kinase activity of RIPK1, and thus inhibit mixed lineage kinase domain-like (MLKL) phosphorylation. We speculate that Nec-1 inhibit NETs release by inhibiting MLKL phosphorylation and perforation. Thus, on the other hand Nec-1 promote neutrophil apoptosis by increasing caspase-3 expression. The roles of Nec-1 inhibiting NETs release and promoting neutrophils apoptosis resolve neutrophilic inflammation.

In addition, we found that Nec-1 also induced apoptosis of neutrophils in BAL and lung tissues (Figures 4K,L, Figure S3). Indeed, we demonstrated that Nec-1 increased the fraction of cleaved caspase-3 positive neutrophils in BAL and lung tissues by immunofluorescence assays (Figures 4K,L). Based on our prior studies, we have proposed a cell death recognition model” for the immune system, in which the consequences of immune responses depend on the type of cell death (50). Necrotic cells aggravate inflammation and upregulate the immune response, while apoptotic cells reduce inflammation and down-regulate immunity (51). During the process of NETosis, intracellular components are released into extracellular space, leading to the presentation of autoantigens to host immune system and the release of damage-associated molecular patterns (DAMPs) that amplify inflammation and immune responses. Thus, the “cell death recognition model” can explain the function of NETs in the pathogenesis of neutrophilic asthma. In contrast, apoptosis, a programmed cell death that maintains membrane integrity and retains toxic neutrophil contents inside the apoptotic bodies, constitutes a mechanism that limits and resolves excessive inflammatory responses. Apoptosis and efficient phagocytosis of apoptotic bodies are crucial in the resolution of inflammation. Multiple studies have demonstrated that delayed neutrophil apoptosis is inherent in neutrophil-dominated airway inflammation (10, 11). Thus, we believe that Nec-1 alleviates inflammation in neutrophil-dominated asthma by inhibiting NETs formation and promoting neutrophil apoptosis, resulting in down regulation of the immune response and reduction the release of inflammatory factors (Figure 6). On the contrary, recently studies indicated that glucocorticoids have no inhibitory effect on NETs formation (52), and found that glucocorticoids can enhance the potential effect of neutrophils by delaying their apoptosis (42) which may explain the poor response to glucocorticoids in patients with neutrophil-dominated asthma.

In conclusion, the current study has shown that NETs have a critical function in the occurrence and aggravation of neutrophil-dominated asthma. Additionally, the results explain the lack of effectiveness of glucocorticoid therapy in neutrophilic asthma. The inhibition of NETs formation by Nec-1 can serve as one of the targets in the treatment of neutrophil-dominated asthma.

## Data Availability Statement

The datasets generated for this study are available on request to the corresponding author.

## Ethics Statement

The studies involving human participants were reviewed and approved by The Third Affiliated Hospital, Southern Medical University. The patients/participants provided their written informed consent to participate in this study. The animal study was reviewed and approved by The Third Affiliated Hospital, Southern Medical University.

## Author Contributions

XH and HJ carried out most of the experiments, participated in the analysis of data, and drafted the manuscript. JHW participated in the design of the study, data analysis and interpretation, and drafted the manuscript. XZ, JZ, and JW participated in the animal experiments and performed the statistical analysis. CY and JH participated in the immunofluorescence assays and carried out the flow cytometry analysis. KL participated in the assessment of histopathological changes and revising the manuscript. ES participated in the design and coordination of the study, and finalized the manuscript. All authors read and approved the final manuscript.

## Conflict of Interest

The authors declare that the research was conducted in the absence of any commercial or financial relationships that could be construed as a potential conflict of interest.

## Supplementary Material

Supplementary Figure 1 **(A,B)** Human neutrophils (5 × 10^6^ cells/ml) were treated with buffer control, PMA (25 nM) with or without different concentrations of Nec-1 (50 or 100 μM) for 2 h. After cell lysis, proteins were subjected to pMLKL. Western Blots are representative of two times of separate experiments.

Supplementary Figure 2 Double-stranded DNA synthesized *in vitro* damage HUVEs. **(A)** Morphological changes of human umbilical vascular endothelial cells for treatment with 500 and 5,000 ng/ml DNA for 12 h. **(B)** LDH release was detected after co-cultured 1 h. **(C)** cell viability was detected by CCK8 for 12 h. The data are shown as mean ± SD. (^*^*p* < 0.05, ^**^*p* < 0.01, ^***^*p* < 0.001, ns, not significant). The results are representative of at least three experiments.

Supplementary Figure 3 Nec-1 could induce neutrophils apoptosis in a dose-dependent manner. **(A,B)** Different concentrations of Nec-1 co-cultured with isolated human neutrophils for 18 h. The fraction of apoptotic neutrophils detected by flow cytometry. The results are representative of three independent experiments. The values were shown as mean ± SEM. (^**^*p* < 0.01, ^****^*p* < 0.0001). **(C)** Representative pictures of flow cytometry were shown.

Supplementary Figure 4 HL-60 could differentiate into mature neutrophils by all trans retinoic acid (ATRA). **(A)** HL-60 was induced by all trans retinoic acid (ATRA) 1 μM for 3 days to differentiate into mature neutrophils, and the nuclei changed from round to paging nuclei. **(B)** Representative immunofluorescence of HL-60 and ATRA induced HL-60 cells stimulated with PMA (25 nM). **(C)** Representative immunofluorescence of HL-60 and ATRA induced HL-60 cells stimulated with PMA (25 nM), SAU (*Staphylococcus aureus*), and A23187(Calcium ion carrier).

Video S1 Indicate Ctrl group.

Video S2 Indicate PMA(25nM) group.

Video S3 Indicate PMA(25 nM) + Nec-1(50µM) group.

Video S4 Indicate PMA(25 nM) + Nec-1(100µM) group.

## Abbreviations

NETs: neutrophil extracellular traps
EETs: eosinophil extracellular traps
Nec-1: necrostatin-1
RIP1: receptor-interacting protein 1
PMA: phorbol 12-myristate 13-acetate
PBNs: peripheral blood neutrophils
LDH: lactate dehydrogenase
OVA: ovalbumin
CFA: complete Freund's adjuvant
BAL: bronchoalveolar lavage
RIP1: receptor-interacting protein 1; kinase
MPO: myeloperoxidase
AHR: airway hyperresponsiveness
PAF: platelet-activating factor
RIPK3/MLKL: receptor-interacting protein 3 kinase/mixed-lineage kinase domain-like protein
DAMPs: damage-associated molecular patterns.
