# Supplementary figures and images for: Necrostatin-1 Ameliorates Neutrophilic Inflammation in Asthma by Suppressing MLKL Phosphorylation to Inhibiting NETs Release
Source: Front Immunol. 2020 Apr 24;11:666. doi: 10.3389/fimmu.2020.00666 (PMC7194114; doi:10.3389/fimmu.2020.00666)

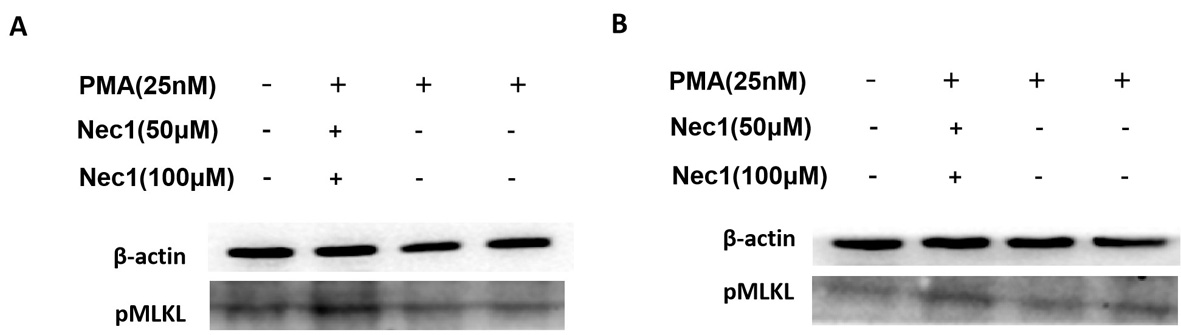

Supplement: Supplementary Figure 1 — (A,B) Human neutrophils (5 × 10^6^ cells/ml) were treated with buffer control, PMA (25 nM) with or without different concentrations of Nec-1 (50 or 100 μM) for 2 h. After cell lysis, proteins were subjected to pMLKL. Western Blots are representative of two times of separate experiments. [file Image_1.JPEG]

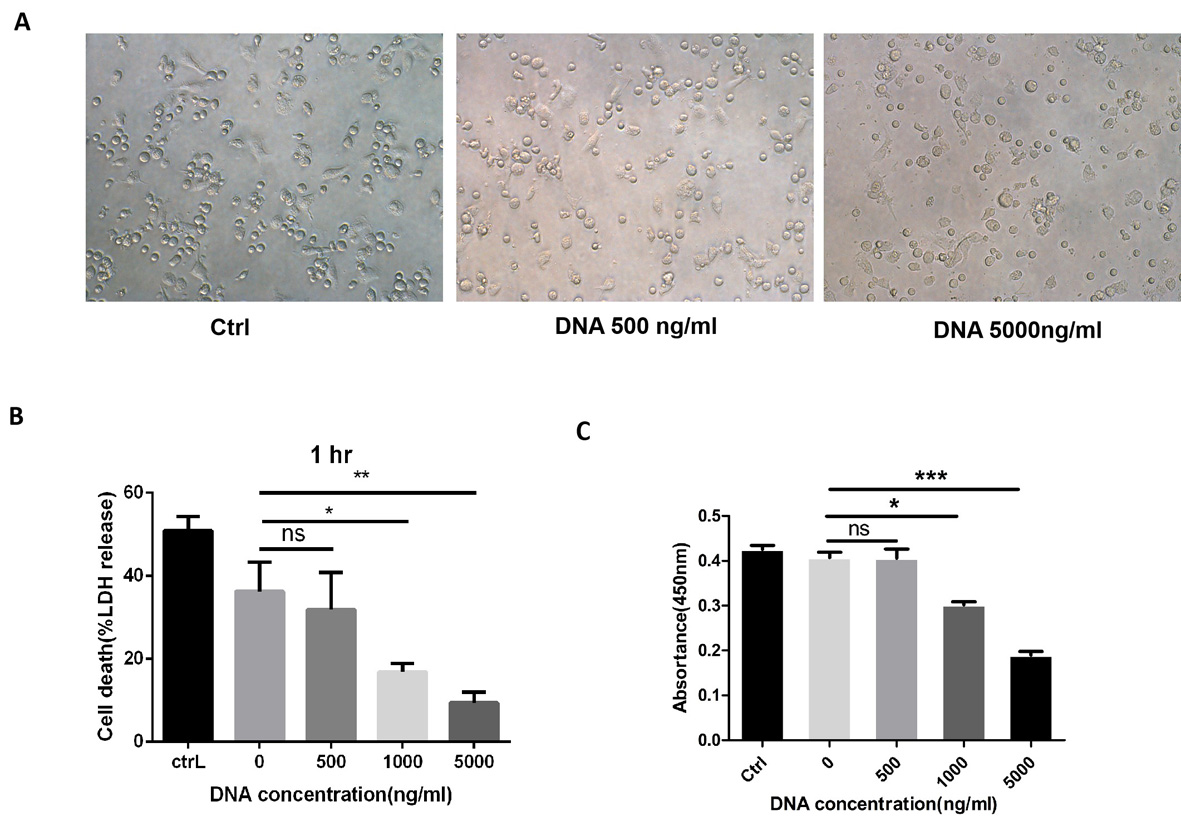

Supplement: Supplementary Figure 2 — Double-stranded DNA synthesized in vitro damage HUVEs. (A) Morphological changes of human umbilical vascular endothelial cells for treatment with 500 and 5,000 ng/ml DNA for 12 h. (B) LDH release was detected after co-cultured 1 h. (C) cell viability was detected by CCK8 for 12 h. The data are shown as mean ± SD. (*p < 0.05, **p < 0.01, ***p < 0.001, ns, not significant). The results are representative of at least three experiments. [file Image_2.JPEG]

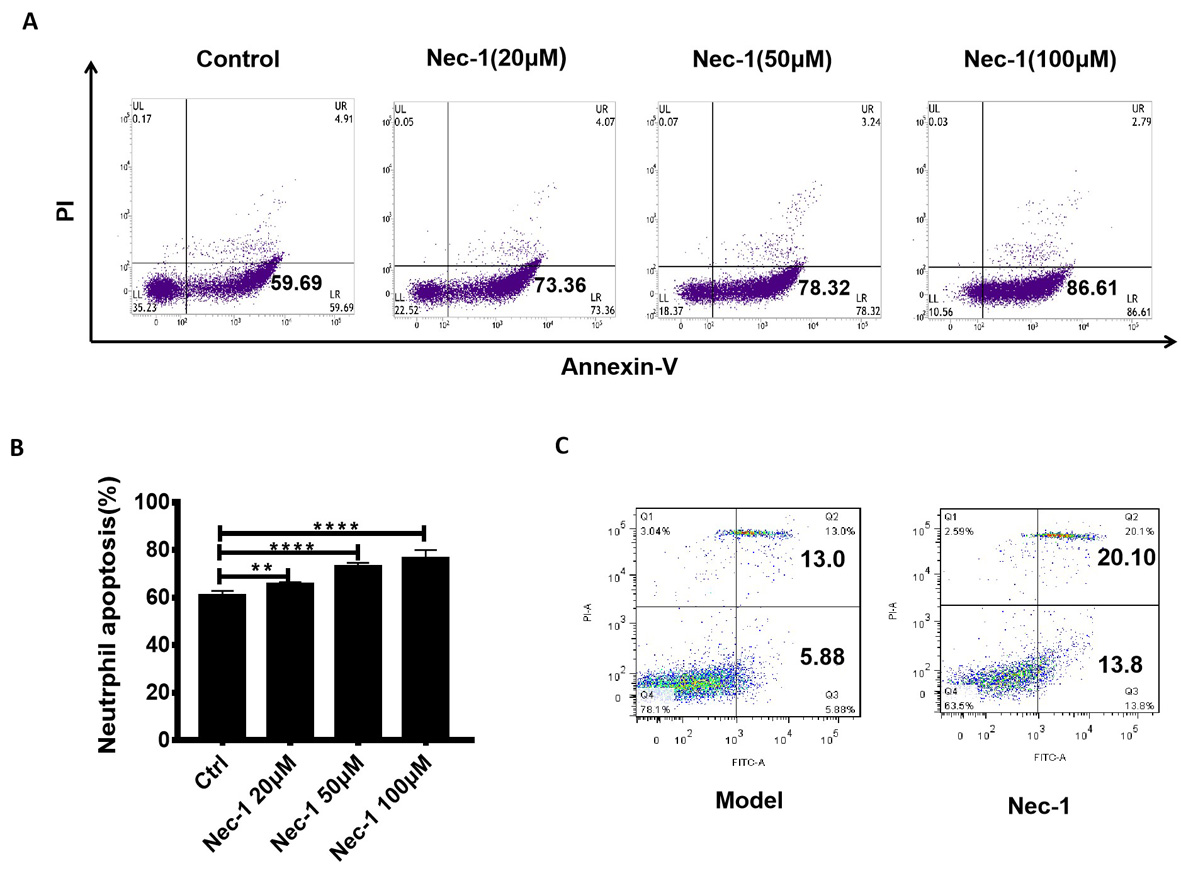

Supplement: Supplementary Figure 3 — Nec-1 could induce neutrophils apoptosis in a dose-dependent manner. (A,B) Different concentrations of Nec-1 co-cultured with isolated human neutrophils for 18 h. The fraction of apoptotic neutrophils detected by flow cytometry. The results are representative of three independent experiments. The values were shown as mean ± SEM. (**p < 0.01, ****p < 0.0001). (C) Representative pictures of flow cytometry were shown. [file Image_3.JPEG]

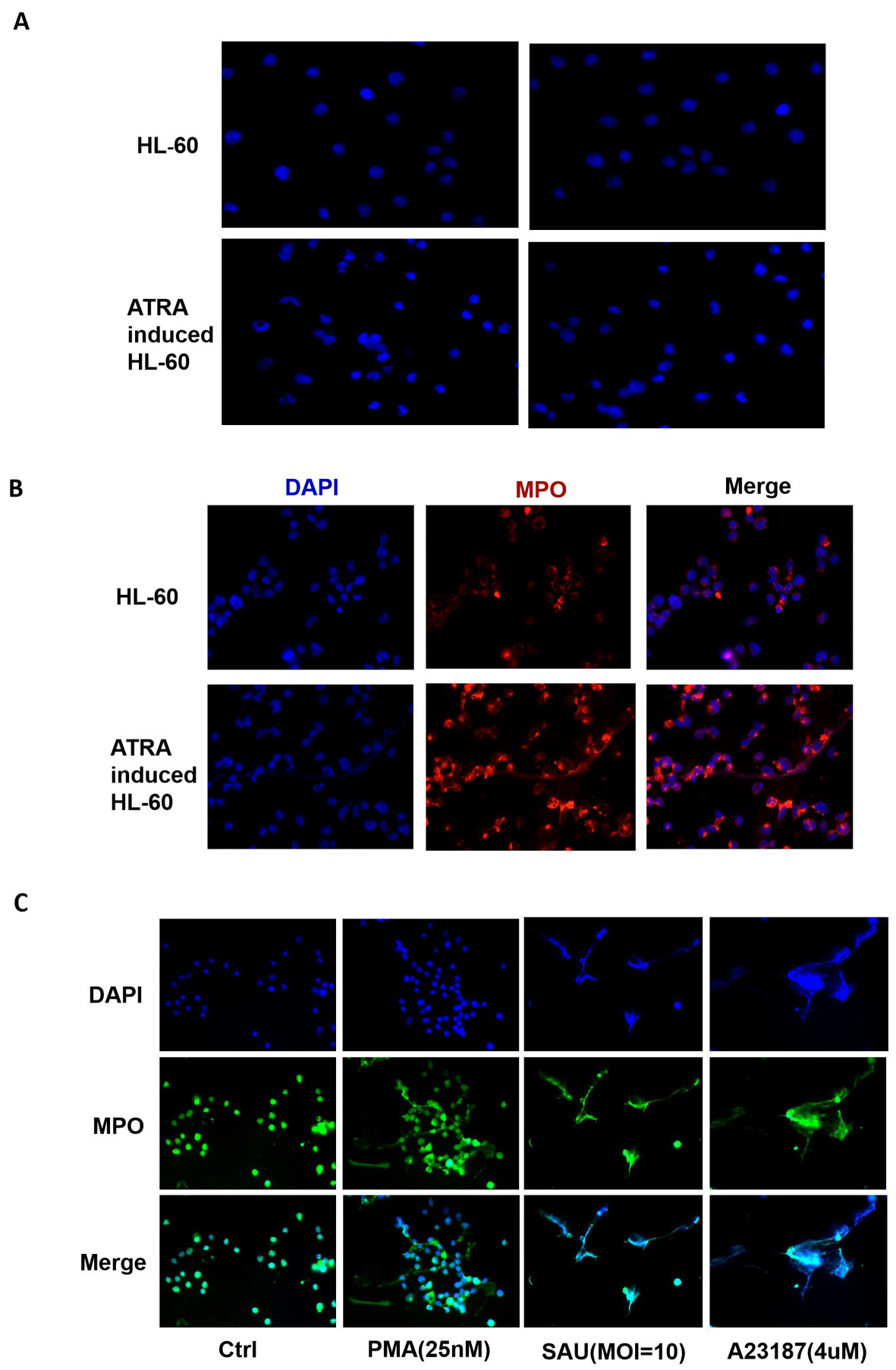

Supplement: Supplementary Figure 4 — HL-60 could differentiate into mature neutrophils by all trans retinoic acid (ATRA). (A) HL-60 was induced by all trans retinoic acid (ATRA) 1 μM for 3 days to differentiate into mature neutrophils, and the nuclei changed from round to paging nuclei. (B) Representative immunofluorescence of HL-60 and ATRA induced HL-60 cells stimulated with PMA (25 nM). (C) Representative immunofluorescence of HL-60 and ATRA induced HL-60 cells stimulated with PMA (25 nM), SAU (Staphylococcus aureus), and A23187(Calcium ion carrier). [file Image_4.JPEG]
